# Origin of the omnipotence of eukaryotic release factor 1

**DOI:** 10.1038/s41467-017-01757-0

**Published:** 2017-11-10

**Authors:** Christoffer Lind, Ana Oliveira, Johan Åqvist

**Affiliations:** Department of Cell & Molecular Biology, Uppsala University, Biomedical Center, Box 596, SE-751 24 Uppsala, Sweden

## Abstract

Termination of protein synthesis on the ribosome requires that mRNA stop codons are recognized with high fidelity. This is achieved by specific release factor proteins that are very different in bacteria and eukaryotes. Hence, while there are two release factors with overlapping specificity in bacteria, the single omnipotent eRF1 release factor in eukaryotes is able to read all three stop codons. This is particularly remarkable as it is able to select three out of four combinations of purine bases in the last two codon positions. With recently determined 3D structures of eukaryotic termination complexes, it has become possible to explore the origin of eRF1 specificity by computer simulations. Here, we report molecular dynamics free energy calculations on these termination complexes, where relative eRF1 binding free energies to different cognate and near-cognate codons are evaluated. The simulations show a high and uniform discrimination against the near-cognate codons, that differ from the cognate ones by a single nucleotide, and reveal the structural mechanisms behind the precise decoding by eRF1.

## Abstract (web summary)

The eukaryotic release factor eRF1 is able to recognize the three stop codons UAA, UAG and UGA with high accuracy, while discriminating against near-cognate codons. Here the authors use molecular dynamic simulation to provide insight into the molecular basis behind the remarkable codon specificity of eRF1.

## Introduction

**Fig. 1:**
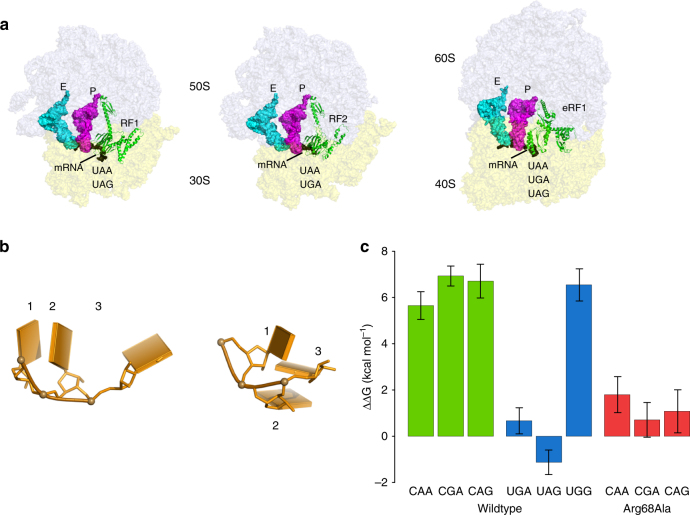
Stop codon recognition in bacteria and eukaryotes. **a** View of bacterial (RF1 and RF2) and eukaryotic release factors (eRF1) bound to their respective ribosomes. The latter is omnipotent and reads all three stop codons. **b** The mRNA stop codon conformation is different in bacteria (left) and eukaryotes (right), where the latter is characterized by an mRNA U-turn. **c** Calculated binding free energy changes associated with single mutations from cognate to near-cognate stop codons (green and red bars for wildtype and mutant eRF1, respectively) and the corresponding binding free energies for the UGA, UAG, and UGG codon relative to UAA (blue bars). Error bars, 1 s.e.m. from at least 15 independent simulations

A major difference in the protein synthesis machinery between bacteria and eukaryotes regard the termination of mRNA translation on the ribosome. While the three stop codons UAA, UAG, and UGA are largely conserved throughout the three kingdoms of life, albeit with notable exceptions^1^, the way they are decoded differs substantially. Hence, in bacteria two class-1 release factors (RFs) with overlapping specificity are generally required for stop codon recognition. Here, RF1 reads the UAA and UAG codons, while RF2 reads UAA and UGA. In eukaryotes and archaea, on the other hand, a single omnipotent RF is able to read all three stop codons^2^. This is quite remarkable since these RFs (eRF1 in eukaryotes and aRF1 in archaea) also apparently can discriminate against the tryptophan codon with high fidelity. That is, the omnipotent RF can recognize three combinations of purines in the second and third codon position (UAA, UAG, UGA), but avoid reading the fourth alternative (UGG) (Fig. 1). How this can be achieved from a structural viewpoint has remained a mystery, but recent cryo-EM structures^3,4^ of eRF1 complexes with the ribosome now hint at a solution of the problem.

When a stop codon is presented at the decoding site, eRF1 binds to the ribosome in ternary complex with the class-2 release factor eRF3 and GTP. The latest cryo-EM structures^5^ reveal that eRF1 first binds in its closed conformation, similar to what has been suggested also for bacterial RFs^6,7^. This is followed by GTP hydrolysis and dissociation of eRF3, whereupon eRF1 accommodates on the ribosome and inserts the universally conserved GGQ motif of its M domain into the A-site of the peptidyl-transferase center (PTC)^5,8^. It is the glutamine sidechain of this motif that coordinates a catalytic water molecule in the PTC and promotes hydrolysis of the ester bond between the P-site transfer RNA and the nascent peptide^9,10^. It should also be noted here that the PTC is located some 75–80 Å away from the decoding site. The structures further show that the mRNA stop codon and the N-domain of eRF1, which is responsible for codon recognition, maintain the same overall conformation in the decoding site throughout the termination process. That is, the detailed structure of the eRF1–codon interaction is virtually identical in the pre-accommodated state and the final state where the M domain has accommodated into the ribosomal A-site^5^.

Although eRF1 and its bacterial counterparts RF1 and RF2 have the same biological function, they are structurally different and have little sequence homology, apart from the universally conserved GGQ motif which is common to all class-1 RFs^11^. Moreover, crystal structures of bacterial termination complexes revealed that the first two bases of the stop codon are stacked, while a histidine sidechain from the RF intercalates between these and the third base of the stop codon^12–14^. This histidine residue is also highly conserved in both RF1 and RF2 (His193 and His202, respectively; *Thermus thermophilus* numbering)^12–14^. However, the stop codon conformation adopted in eukaryotic ribosome complexes with eRF1 is distinctly different. Here, the stacked conformation of the first and second bases is lost and the codon instead presents an unexpected U-turn conformation^3,4^ (Fig. 1). This conformation has a more compact shape and is clearly different from the standard stacked shape of sense codons. Before the emergence of 3D structural data, the codon specificity of eRF1 was biochemically mapped to highly conserved amino-acid motifs in its N-domain. Photochemical cross-linking experiments suggested that the TASNIKS motif (residues 58–64) is crucial to recognize the invariant first position uracil^15^, while the GTS loop (residues 31–33) and the YxCxxxF motif (residues 125–131) could be associated with reading of the second and third stop codon positions^16–19^. The highly conserved Glu55 has also been shown to be prominent for correct stop codon recognition^17^. In addition to the biochemical and mutagenesis studies that have attempted to determine the key residues for recognition at the different stop codon positions^16,17,20–25^, the ribosome-bound structures of eRF1^3,4^ now clearly provide a breakthrough for understanding the eRF1-stop codon recognition.

The two structural studies of eRF1 complexes^3,4^ both point to the importance of Lys63 for recognizing the first position uracil and also suggest that Glu55 is the key to discriminating against a double guanine pair in the last two codon positions. It was thus hypothesized that Glu55 could introduce unfavorable repulsions with a putative GG pair, thereby disturbing the hydrogen bonding network required for proper stop codon recognition^3,4^. However, while Beckmann and co-workers^4^ emphasize a direct sidechain contact between Cys127 of the YxCxxxF motif and the second codon base, the Ramakrishnan group structure^3^ rather suggests that the Cys127 main chain interaction with the rRNA base A1825 is important for stacking of the two last bases in the codon U-turn. The two structures further agree with each other regarding the interaction between the GTS loop and third position nucleotide. This loop is described as flexible with the ability to adopt different conformations depending on which purine nucleotide is to be sensed^3,19^. In this context, it is also interesting to look at exceptions from the standard coding of termination. For example, in two ciliate species *Paramecium* and *Stylonychia* the release factor only exhibits UGA decoding specificity^26^, while UAA and UAG are sense codons^27^. *Paramecium tetraurelia* is of particular interest here since the highly conserved Glu55 and Thr58 residues are mutated to Asn and Glu, respectively, although Cys127 remains invariant.

While 3D structures of ribosome complexes yield invaluable information for formulating reasonable mechanistic hypotheses regarding function, it is often not so straightforward to actually validate or invalidate such proposals by experimental means. This also holds true for the above hypotheses for how eRF1 is able to achieve its peculiar codon specificity. Due to the low resolution of electron density for the codon binding site in the N-domain, a clear atomistic understanding of the structural basis of stop codon recognition is still lacking. For instance, the density for the hypothesized crucial Glu55 sidechain is completely missing^3,4^. In this work, we have computationally investigated the principles by which eRF1 recognizes each individual stop codon while effectively discriminating against the near-cognate sense codons CAA, CGA, CAG, and UGG. Here, computer simulations can play a very important role in bridging between structure and biochemistry, since the detailed energetics of molecular recognition processes can nowadays be evaluated with sufficiently high fidelity. That is, with a typical free energy calculation accuracy of about ±1 kcal mol^−1^, cognate vs. non-cognate codon binding can be reliably quantified. Hence, we use molecular dynamics (MD)-based free energy calculations to explore the energetics and structural origin of stop codon recognition by eRF1. The relative eRF1 binding free energies for the different stop codons show that there is little variation in binding strength between these. In contrast, there are large energetic penalties associated with binding to different sense codons, including the tryptophan codon UGG. The simulations further allow identification of specific amino-acid interaction patterns depending on which codon is positioned in the decoding site.

## Results

### Molecular dynamics simulations and free energy perturbation

Free energy perturbation method (FEP) calculations^28^ were performed utilizing the cryo-EM structures of the mammalian release factor (eRF1) complexes with the three stop codons UAA, UAG, and UGA (PDB accession numbers 3JAG, 3JAH, and 3JAI, respectively)^3^. To evaluate the relative free energies of binding to different codons single-nucleotide mutations were performed, starting from each of the different stop codons. These calculations were carried out both with and without eRF1 bound to the ribosome, which allows the relative binding free energies to be evaluated by a standard thermodynamic cycle^29^. All calculations were repeated 15 times with different initial velocities in the MD simulations.

### Reading of the first codon position

**Fig. 2:**
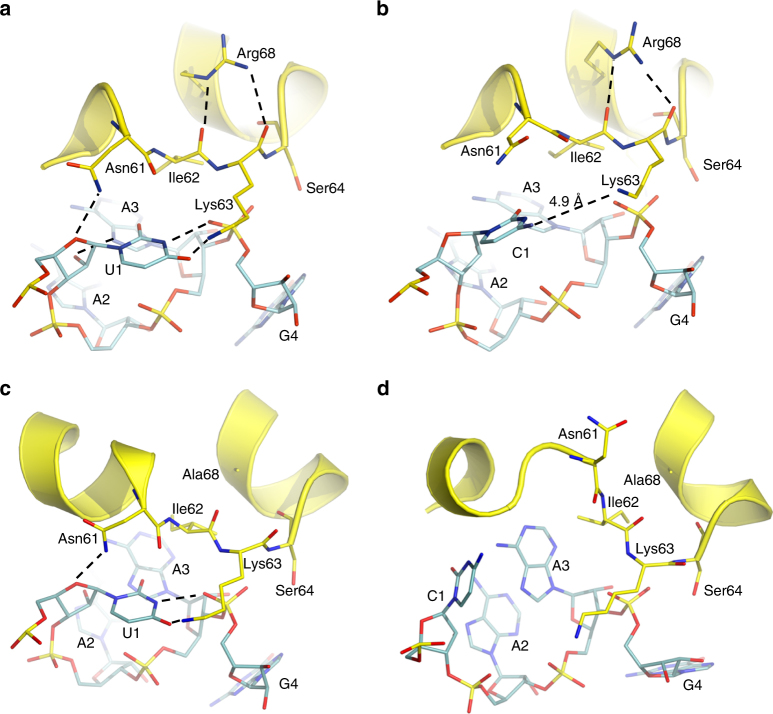
Reading of the invariant first position uracil. **a** Average MD structure showing the favorable interactions of the first position uracil (UAA codon) with Lys63 of the NIKS loop and an additional hydrogen bond to the phosphate backbone of the +4 nucleotide. **b** With a first position cytosine, the repulsion with Lys63 leads to loss of hydrogen bonding and an increased distance between the base and this eRF1 sidechain. **c** Average MD structure of the Arg68Ala mutation with the UAA codon bound, which is similar to the corresponding wild-type structure. **d** The non-cognate CAA codon is predicted to induce a sizeable conformational change of the NIKS loop in the Arg68Ala mutant, where the stabilizing arginine interaction with the NIKS backbone is missing

Uracil occupies the first nucleotide position in all standard stop codons. The specificity for this nucleotide should thus be a requirement for initiating the termination process. To evaluate the energetic penalty against the seemingly near-cognate CAA, CAG and CGA (glutamine and arginine) codons, we mutated the first position in all three stop codons in the free energy simulations. The results clearly show a large and uniform discrimination against these three codons. The binding free energies of eRF1 to the three sense codons CAA, CAG and CGA, relative to the stop codons differing by a single mutation, are thus all predicted to be about 6 kcal mol^−1^ (Fig. 1c). This corresponds to more than a factor of 10^4^ in terms of binding affinity, demonstrating the high specificity of eRF1 for the first position uracil. The high bias toward uracil reading (Fig. 2a) is indeed found to be largely caused by the repulsive interactions between the Lys63 sidechain and the amine of the cytosine base in the sense codons (Fig. 2b), as indicated by the cryo-EM structures^3,4^. Consequently, the cytosine becomes dislocated from the compact U-turn structure observed with cognate stop codons. The cryo-EM structures differ with regard to the rotamer of the cognate uracil base and in all our MD simulations the nucleobase initially rotates to the conformation observed in ref. ^4^, indicating that this is the preferred rotamer.

As had been suggested by cross-linking experiments^15^, and now supported by the structural data^3,4^, the NIKS loop is in close proximity to the first codon position. Here, the O4 atom interacts with the amine group of Lys63 of the NIKS motif and N3 makes an additional hydrogen bond to the mRNA phosphate group at position +4 (the first position U is denoted +1). This conformation allows the first position U to satisfy two hydrogen bonds as in a U–A Watson–Crick pair with donor–acceptor distances of 3.0 and 2.9 Å, respectively (Fig. 2a). Similar interactions involving N3 and O4 hydrogen bonds were also found in earlier work on both bacterial and mitochondrial termination complexes^29,30^. The second O2 oxygen atom, which is present in both uracil and the cytosine, shows no interaction with eRF1 in the MD simulations. The Asn61 sidechain of eRF1 further stabilizes the stop codon conformation by interacting with the sugar base of the first nucleotide. Moreover, the 2′-oxygen of U + 1 hydrogen bonds to N7 of the third codon base, which again stabilizes the U-turn, but neither of these two interactions contribute to discrimination since they do not involve the first position nucleobase itself. In fact, the NIKS loop appears very stable and rigid during the MD simulations, both with sense and stop codons bound, which presumably contributes to its role in codon discrimination. This is evidenced by the evolution of its backbone root mean square deviation (RMSD) from the corresponding experimental starting structures during the free energy simulations. The NIKS loop RMSD thus remains below 0.6 Å during all of the FEP mutations and the average over all replicate simulations of the eRF1 complexes (135 in total) is only 0.38 Å, indicating that the loop is not able to remodel its conformation in response to these codon changes. Furthermore, the highly conserved Arg68 residue^31^ appears crucial for maintaining the NIKS loop conformation and two hydrogen bonds between its sidechain and the backbone of Ile62 and Lys63 could be identified (Fig. 2). This finding agrees well with the loss of termination efficiency observed experimentally for the Arg68Ala mutation^25^. To further investigate the NIKS loop stability and the role of Arg68 in stabilizing the NIKS loop, we mutated Arg68 to alanine and performed free energy calculations on the ability of this eRF1 mutant to discriminate against a cytosine in the first codon position. These calculations predict a remarkable loss of fidelity, where the penalty for reading a cytosine now drops to 1–2 kcal mol^−1^ (Fig. 1c). Examination of the average structure from these simulations further reveals that the NIKS loop undergoes large conformational changes with non-cognate codon bound, as a response to the missing backbone interactions with Arg68 (Fig. 2c, d).

### Codon recognition at the second and third positions

To evaluate the binding specificity at the last two codon positions, we first carried out free energy perturbation calculations of the synonymous stop codon mutations from UAA to UGA and UAG and vice versa. These mutations show that eRF1 has little or no preference for any of the three stop codons, in agreement with kinetic data for human eRF1^27^ (Fig. 1). The calculated binding free energies of eRF1 to the UGA and UAG stop codons relative to UAA are thus both near-zero, ${\mathrm{\Delta \Delta }}\rm G_{{\mathrm{bind}}} = 0.7$ and −1.1 kcal mol^−1^ for UGA and UAG, respectively (Fig. 1). In contrast, the important and non-trivial feature of a high discrimination against the tryptophan codon UGG is clearly seen from the simulations. Thus, the energetic penalty against reading UGG is predicted by the calculations to be very high, ${\mathrm{\Delta \Delta }}\rm G_{{\mathrm{bind}}} = 6.6$ kcal mol^−1^ relative to UAA. Hence, the picture emerging from these calculations is a high and uniform penalty of about 6 kcal mol^−1^ for all the four non-cognate codons examined. This is in agreement with kinetic measurements in a yeast termination assay, where more than a 10^3^-fold difference in $k_{{\mathrm{cat}}}{\mathrm{/}}K_{\mathrm{M}}$ for peptide release on the UAA and UGG codons is observed (Prof. M. Ehrenberg, personal communication).

**Fig. 3:**
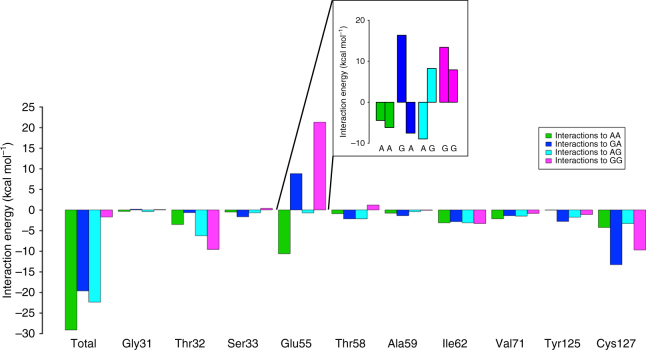
Interaction energies between the codon bases and their surroundings. Average non-bonded interaction energies (electrostatic and van der Waals) between the last two codon bases and key eRF1 residues around the codon are shown. Green, blue, cyan, and pink bars denote the UAA, UGA, UAG, and UGG codons, respectively. The critical interactions with Glu55 are further divided into their second and third base components in the inset, where the two bars of equal color denote interactions with these two bases, respectively, for each codon

**Fig. 4:**
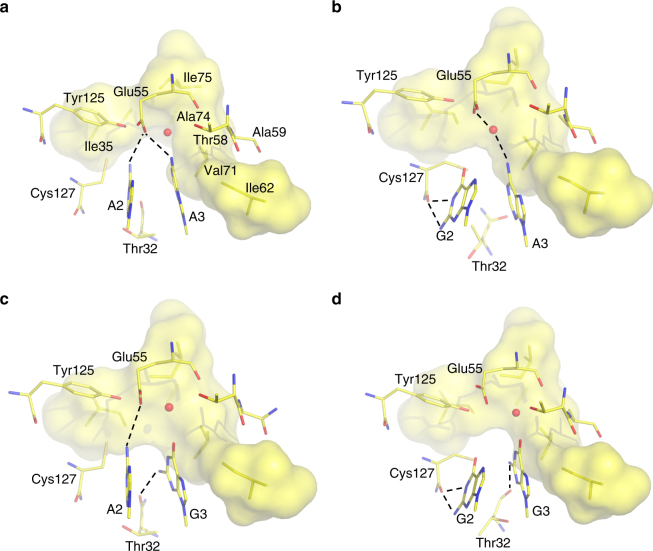
Structural basis of second and third position stop codon reading. Average MD structures of eRF1 bound to the **a** UAA, **b** UGA, **c** UAG, and **d** UGG codons. The hydrophobic cluster of residues (Ile35, Ile62, Val71, Ala74, Ile75, and Cys127) is shown as a transparent surface and key water molecules are depicted as red spheres. Key hydrogen bonds involved in base recognition are indicated as dashed lines

To investigate the proposal^3,4^ that the negatively charged Glu55 sidechain is responsible for discriminating solely against UGG among the four UNN codons, we examined the behavior of Glu55 during the MD simulations. This residue turns out to be remarkably stable and essentially maintains the same conformation regardless of which codon is present (Supplementary Fig. 1). However, the interaction energetics between eRF1 and the last two codon positions turns out to be very informative. We evaluated the average pairwise non-bonded interaction energies between these codon positions and key eRF1 residues that have been implicated from mutagenesis experiments to be crucial for correct codon reading^16,19,22,25^ (Fig. 3). While the total non-polar contribution to these interaction energies is essentially constant among all four calculated codons, it is the polar interactions that give distinct differences, which explain the origin of the eRF1 omnipotence in stop codon reading. Importantly, it can be seen that the three cognate codons all have large favorable (negative) total interaction energies with the 10 eRF1 residues surrounding the second and third codon bases. The arrangement of these residues surrounding the codon is shown in Fig. 4. However, when the UGG codon is bound the overall interaction is considerably less favorable (Fig. 3). This effect is indeed entirely due to electrostatic repulsion between the Glu55 sidechain and the double guanine pair in UGG. In the case of a UAA codon, the AA interaction with Glu55 is about 10 kcal mol^−1^ negative, with about equal attractive contribution from the two bases. In the case of UAG, the Glu55 attraction with the second position is more or less balanced by its repulsion with the third. However, the Glu55 repulsion is about twice as strong with a G in the second position compared to the third, making its overall interaction with UGA unfavorable. The situation with UGA is, however, saved by the fact that Cys127 has a distinctly favorable backbone interaction with a second position G, which more than compensates for the unfavorable Glu55 interaction. Since Glu55 strongly prefers A’s in both of the last codon positions, the guanine penalties add up to a large repulsion with UGG and, in this case, the Cys127 interaction is not enough to compensate the energetic penalty. Hence, the distinct discrimination against UGG can, at least qualitatively, be explained in terms of a fine-tuned energetic interplay predominantly between Glu55 and Cys127. We say qualitatively, since the energy components discussed are only enthalpic contributions and entropic effects associated with these interactions are not included in Fig. 3.

The residue Cys127 is also highly conserved in eRF1 and aRF1 and has been suggested from experiments to be pivotal for correct codon recognition^22,25^. Our MD simulations show that this residue, or rather its backbone, is particularly selective for a second position guanine, with which it makes double hydrogen bonds (Fig. 4b, d). The strong conservation of Cys127 is likely due its bulky sulfur atom being sequestered between the codon and the also highly conserved residues Ile35 and Tyr125. This appears to enforce a backbone conformation at the tip of β-strand 4, with the Cys127 carbonyl group in appropriate orientation for sensing the second nucleotide position. Its carbonyl has the capability of hydrogen bonding with both the N1 and N2 atoms of guanine and these interactions are lost with an adenine in the second codon position, which is also reflected by the interaction energetics in Fig. 3. Besides its direct interaction with second codon position, the Cys127 sidechain forms a hydrophobic cluster together with Ile35, Ile62 (of the NIKS motif), Val71, Ala74, and Ile75 around the third codon base (Fig. 4). This cluster is dispersed along the eRF1 amino-acid sequence and has therefore not been identified as a unique motif, but it is clear that it forms a distinct structural motif that is highly conserved among the eRF1 sequences^31^. Hence, both Ile62 and Cys127 would perhaps be better described as part of this hydrophobic motif than of the NIKS and YxCxxxF motifs to which they are usually assigned. Due to the packing of this sidechain cluster around the edge and (downstream) face of the third base, it prevents entry of excessive solvent that could otherwise compensate for missing hydrogen bonds. Thus, the role of the hydrophobic cluster clearly appears to be partial desolvation of the third codon position. It is therefore likely that all of the cluster members (Ile35, Ile62, Val71, Ala74, Ile75, and Cys127) make small hydrophobic contributions to the codon selectivity. In order to test this hypothesis, we mutated Cys127 to glycine and repeated the FEP calculations of the UAA → UGG codon mutation. These calculations predict the Cys127Gly mutation to reduce the penalty against UGG from 6.6 to 3.9 kcal mol^−1^, with accompanying water penetration, which shows that the hydrophobic cluster indeed is critical. As far as the YxCxxxF motif is concerned, the hydroxyl group of Tyr125 interacts with Glu55, which assists in stabilizing the hydrogen bond with the amine group in a second position adenine. The conserved GTS loop does not provide any large contributions to codon binding, with the exception of a hydrogen bond between the Thr32 backbone carbonyl and a third position G. This favorable interaction is also seen from the energetics in Fig. 3.

## Discussion

While the common stop codons UAA, UGA, and UAG are used in the genetic codes of most species, the mechanisms for recognizing them are diverse. In bacteria, two RFs with overlapping specificities are required to terminate mRNA translation and yield the correct protein product. In eukaryotes, on the other hand, the single eRF1 release factor is able to read all three stop codons. Numerous studies of eRF1 mutants have investigated the principles behind its omnipotence, or triple codon reading ability, but it is only with the emergence of 3D structures of termination complexes that quantitative structure–function relationships can be established.

Our molecular dynamics free energy perturbation calculations show that eRF1 has a common pattern of binding to all three stop codons and that this reflects a very specific interplay of amino acids within the codon reading domain. The simulations show that the first position uracil consistently has its O4 and N3 hydrogen bonds satisfied, just as in a standard A–U base pair, and that it is the rotated base conformation^4^ that permits this. Hence, in addition to Lys63 it is the +4 phosphate group of the mRNA that holds the uracil in place in its special U-turn conformation (Fig. 2). The presence of a charged residue at position 63 indeed appears to be crucial for efficient recognition of the first stop codon position, as the Lys63Arg mutation is the only one observed experimentally at this position to retain termination efficiency^25^. It should, however, be noted that Lys63 becomes post-translationally hydroxylated at the delta carbon (C4). This modification has been shown to increase the efficiency of eRF1 termination, albeit by less than a factor of two^32^. The modification was thus not modeled here as it is not present in either of the cryo-EM structures used, but it seems likely that the role of it is to further stabilize the Lys63 conformation for interaction with the mRNA. Furthermore, we predict the conformation and stability of the NIKS loop to be affected by the highly conserved Arg68 residue that interacts with the loop backbone. Removal of these interactions by the Arg68Ala mutation is predicted to render the NIKS loop more flexible and to yield a substantial drop in discrimination against a non-cognate first position cytosine.

The first position uracil can thus be said to be read separately from the last two nucleotides and the principal challenge for eRF1 is to efficiently read only the correct combinations of these nucleotides from the four different possibilities. There is thus a notable difference from how bacterial release factors read the stop codons, in which case each nucleotide is recognized individually^29^. That is, the exclusion of the tryptophan UGG codon arises from the strict specificity of RF1 and RF2 for an adenine in the second and third positions, respectively. By forcing the mRNA U-turn conformation, eRF1 will essentially read the last two purine nucleotides as a block unit rather than individually. The hypothesis from the cryo-EM structures^3,4^ is that the negatively charged Glu55 would prevent reading of a double guanine pair due to electrostatic repulsion with the two O6 oxygens, although electron density for its sidechain is missing. This indeed turns out to be true as judged from our simulation energetics, but the fine-tuning behind this phenomenon is quite remarkable. That is, while the favorable Glu55 interactions with second and third position adenines are similar, the repulsion with guanine is considerably stronger in the second position as the guanine dipole moment in that case is oriented antiparallel to the Glu55 electric field, while that of a third position guanine is more perpendicular (Fig. 4d). The favorable interaction between Cys127 and a second position guanine partly compensates for this repulsion but, since the interactions are more or less additive, it is only the GG pair that results in a large net discrimination (Fig. 3). The story is, however, not that simple since solvent molecules could potentially easily compensate for unsatisfied hydrogen bonds and here the conserved hydrophobic sidechain cluster (Ile35, Ile62, Val71, Ala74, Ile75, and Cys127) apparently protects, particularly the second base, against such interactions. Hence, just as in the case of normal mRNA decoding, where the monitoring bases (A1492 and A1493 in bacteria) have been shown to boost discrimination against non-cognate codons by desolvation^33^, we see the same effect here exerted by the hydrophobic cluster.

Taken together, our MD free energy calculations show that the eRF1 discrimination against the near-cognate sense codons CAA, CGA, CAG, and UGG is large (~6 kcal mol^−1^) and uniform. The simulations further show that eRF1 binding to the three stop codons is also characterized by a uniform binding affinity with little energetic preference for any particular codon. The interplay between different amino acids of eRF1 is clearly important for correct codon reading and is what underlies its ability to read three out of four purine–purine combinations. Hence, the binding is largely monitored by two key residues that are universally conserved, Glu55 providing repulsion and Cys127 providing attraction to the critical stop codon guanines. Interestingly, the calculations also predict that eRF1 has a larger discrimination against sense codons than the bacterial counterparts^29^, which may reflect an overall higher degree of evolutionary optimization of its structure.

## Methods

### Molecular dynamics simulations

MD simulations and FEP^28^ were performed using the cryo-EM structures of the mammalian release complex programmed with the three stop codons UAA, UAG, and UGA (PDB accession numbers 3JAG, 3JAH, and 3JAI, respectively)^3^. The MD simulations utilized spherical boundary conditions with a 30 Å radius sphere centered on the N6 atom of the third position nucleotide of the UAA-bound structure, which is the geometrical center of the stop codon in its U-turn conformation. The system was solvated with TIP3P water molecules and solvent molecules close to the sphere boundary were subjected to radial and polarization restraints according to the SCAAS model^34^. Atoms located further than 22 Å away from the sphere center were harmonically restrained to their initial coordinates with a 10 kcal mol^−1^ Å^−2^ force constant. By scaling down distant RNA phosphate charges and neutralizing charged amino acids close to the boundary, the system was kept overall neutral, as described elsewhere^35^. The system was stepwise heated up to the final temperature of 310 K, initially using a 1 fs time step, which was increased to 2 fs during the end of the relaxation and subsequently used for the data collection phase of the free energy calculations. A direct cutoff of 10 Å was employed for all non-bonded interactions except for the atoms directly involved FEP transformations. Electrostatic interactions beyond the 10 Å cutoff were treated with the local reaction field multipole expansion method^36^.

### Free energy calculations

To calculate the relative free energy of binding of eRF1 to different codons, we used the FEP method to introduce mutations from the cognate stop codons to either near-cognate sense codons or to a synonymous stop codon. From the 3JAG structure with UAA-bound transformations to the CAA, UGA and UAG codons were made. Similarly, the 3JAH complex with UAG was used for codon transformation to CAG, UAA and UGG and the 3JAI structure with UGA bound for codon mutations to CGA, UAA and UGG. To obtain the relative eRF1 binding free energies to different codons, the simulations were carried out with and without the bound release factor in accordance with a standard thermodynamic cycle^37^. All FEP calculations used 51 different sampling windows and the calculations were repeated 15 times, yielding a minimum of 30 ns of data collection per transformation. Each repeat was initiated using randomized initial conditions generated from the Maxwell–Boltzmann velocity distribution. All MD simulations and free energy calculations were performed using the CHARMM36^38,39^ force field implemented to the Q molecular dynamics software^40^. Since experimental structures were available for all three cognate stop codons, transformations between these were carried out in both the forward and reverse directions, starting from the relevant cryo-EM structure. Standard errors of the mean are very similar for mutations carried out only in the forward direction compared to those for which the reverse path could also be initiated from an experimental structure (Fig. 1c). Mutations to the UGG codon were started from both UAG and UGA by transforming the second or third adenine, respectively, to guanine and the binding free energy relative to UAA was calculated by averaging the results from the independent two thermodynamic cycles.

### Code availability

Source code for the Q program^40^ is available at https://github.com/qusers/Q6, or upon request from the corresponding author.

### Data availability

The data that support the findings of this study are available from the corresponding author upon request.

## Electronic supplementary material

Supplementary Figure 1
