# Supplementary material for: Origin of the omnipotence of eukaryotic release factor 1
Source: Nat Commun. 2017 Nov 10;8:1425. doi: 10.1038/s41467-017-01757-0 (PMC5681627; doi:10.1038/s41467-017-01757-0)
Supplement: Supplementary file 1 — Supplementary Figure 1 [file 41467_2017_1757_MOESM1_ESM.pdf]

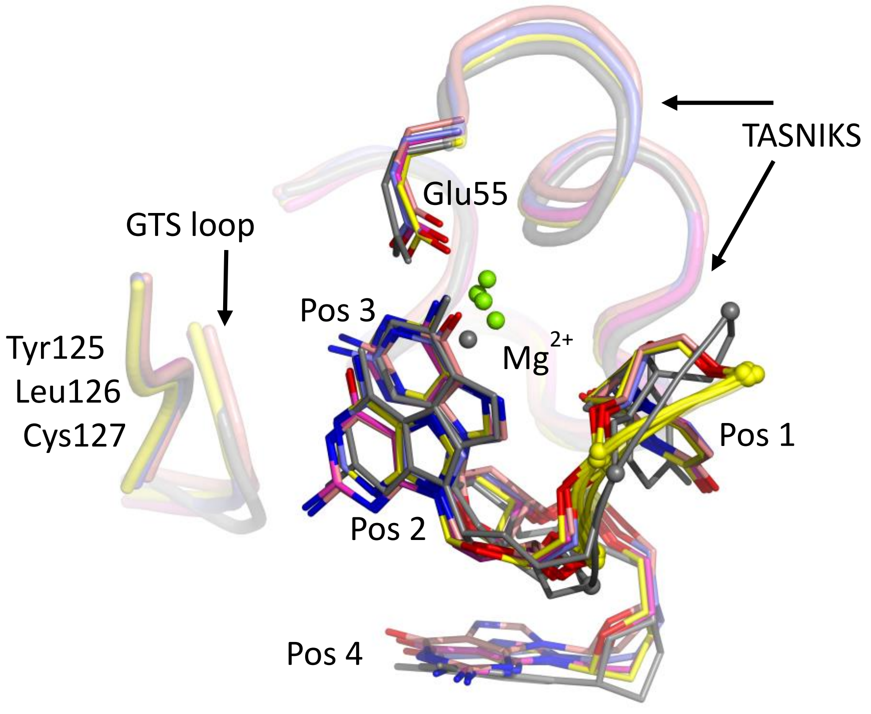

Supplementary Figure 1. **Conformational stability of the conserved eRF1 residue Glu55.** Average MD structures from all MD simulations with the UAA (yellow carbons), UGA (magenta), UAG (blue) and UGG (pink) codons overlayed on the cryo-EM structure with UGA (grey). The stop codons, the +4 nucleotide and Glu55 are shown in stick representation, while surrounding parts of eRF1 are shown as a backbone trace. Green and grey spheres denote magnesium ions from MD and cryo-EM, respectively.
